# Fast Response Solar-Blind Photodetector with a Quasi-Zener Tunneling Effect Based on Amorphous In-Doped Ga_2_O_3_ Thin Films

**DOI:** 10.3390/s20010129

**Published:** 2019-12-24

**Authors:** Mingzhi Fang, Weiguo Zhao, Feifei Li, Deliang Zhu, Shun Han, Wangying Xu, Wenjun Liu, Peijiang Cao, Ming Fang, Youming Lu

**Affiliations:** Shenzhen Key Laboratory of Special Functional Materials, Guangdong Research Center for Interfacial Engineering of Functional Materials, College of Materials Science and Engineering, Shenzhen University, Shenzhen 518000, China; fmzmarcus@163.com (M.F.); 13172464115@163.com (W.Z.); szulff@163.com (F.L.); wyxu@szu.edu.cn (W.X.); liuwj@szu.edu.cn (W.L.); pjcao@szu.edu.cn (P.C.); m.fang@outlook.com (M.F.); ymlu@szu.edu.cn (Y.L.)

**Keywords:** amorphous InGaO thin films, solar-blind photodetector, fast response, quasi-Zener tunneling effect

## Abstract

A high-performance solar-blind photodetector with a metal–semiconductor–metal structure was fabricated based on amorphous In-doped Ga_2_O_3_ thin films prepared at room temperature by radio frequency magnetron sputtering. The photodetector shows a high responsivity (18.06 A/W) at 235 nm with a fast rise time (4.9 μs) and a rapid decay time (230 μs). The detection range was broadened compared with an individual Ga_2_O_3_ photodetector because of In doping. In addition, the uneven In distribution at different areas in the film results in different resistances, which causes a quasi-Zener tunneling internal gain mechanism. The quasi-Zener tunneling internal gain mechanism has a positive impact on the fast response speed and high responsivity.

## 1. Introduction

Ultraviolet light (UV) is electromagnetic radiation, which can be classified into three wavebands: ultraviolet A (UVA, 320–400 nm), ultraviolet B (280–320 nm), and ultraviolet C (UVC, 200–280 nm). UVC light, also known as solar-blind ultraviolet light, is completely absorbed by ozone in the atmosphere and does not exist on the surface of the earth [1,2]. Compared with other light detection technologies, solar-blind ultraviolet light detection technology is less affected by the external environment; the interference of other signal sources on the surface is smaller, and it can work around the clock. Therefore, solar-blind photodetectors based on semiconductor materials with wide-bandgaps, such as AlGaN [3], MgZnO [4], diamond [5,6], and Ga_2_O_3_ [7], have received much attention for applications in missile warning, flame sensors, air purification, space communication, and ozone-layer monitoring. Among the materials, MgZnO and AlGaN exhibit serious composition fluctuations or phase segregation. Additionally, the cost of diamond is too high to apply in practical applications [8,9]. As a direct wide-bandgap semiconductor material, Ga_2_O_3_ has a bandgap width of ~4.9 eV, which is very suitable for solar-blind UV detection [10,11,12], avoiding the complexities and difficulties of fabricating alloys, such as AlGaN and ZnMgO. In addition, Ga_2_O_3_ has great thermal and chemical stability and is inexpensive. Therefore, in recent years, Ga_2_O_3_-based deep ultraviolet light detectors have been widely studied.

Ga_2_O_3_ consists of five different crystal structures. Monoclinic β-Ga_2_O_3_ is the most stable among these structures, and its UV detection properties have drawn increasing attention [13,14]. In 2007, Oshima et al. first reported the metal–-semiconductor–metal (MSM)-type β-Ga_2_O_3_ thin film detector prepared on a sapphire substrate by the molecular beam epitaxy (MBE) method [15]. However, high-quality β-Ga_2_O_3_ thin film materials demand, for example, a high temperature during fabrication, but high-temperature manufacturing conditions are not suitable for flexible devices [16,17]. In addition, due to the persistent photoconductive effect, the speed of the device response and recovery is greatly reduced even to a level of seconds [18]. Recently, amorphous solar-blind photodetectors have demonstrated fast response speeds and simple processing conditions. In 2017, Shujuan Cui et al. fabricated an amorphous Ga_2_O_3_ (a-Ga_2_O_3_) photodetector with a responsivity of 0.91 A/W and a fast decay time of 19.1 μs [19]. At the same time, the amorphous film and room temperature promoted the preparation of a flexible device. However, the responsivity needs to be improved, and the response mechanism also needs to be further researched.

Doping is a good method to help change the photoelectric properties of a device. In_2_O_3_ is a very important n-type semiconductor with a bandgap of 3.6–3.75 eV and has high conductivity and high transmittance in the visible light region. At the same time, In_2_O_3_ also has excellent chemical and thermal stability and is widely used in many fields due to its unique excellent photoelectric properties [20]. In_2_O_3_ and Ga_2_O_3_ can be properly combined into a new InGaO (IGO) oxide alloy with an adjustable bandgap width between 3.6–4.9 eV, thus the detection range of a photodetector could be broadened.

In this paper, we demonstrated the fabrication of an amorphous In-doped Ga_2_O_3_ (a-IGO) thin film MSM photodetector on a sapphire substrate with both high responsivity and fast response speed. We found that In doping leads to the bandgap change, which is the reason for the larger detection range. The photoelectrical characteristics and mechanisms of the fabricated devices were also investigated.

## 2. Materials and Methods

**Figure 1:**
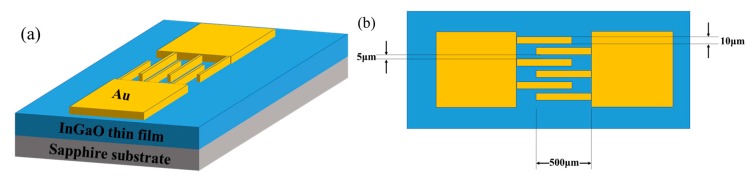
(**a**) The device diagram and (**b**) the finger diagram of the a-IGO thin film solar-blind photodetector.

The a-IGO thin films were grown on c-plane sapphire substrates by radio frequency (RF) magnetron sputtering, and the sputtering target was an IGO ceramic target (Ga:In = 5:2 at%). The sapphire substrates were cleaned in acetone, ethanol and deionized water for 45 min using an ultrasonic cleaning machine. The chamber base pressure was maintained at 5 × 10^−4^ Pa. The sputtering process was carried out for 35 min with a working pressure of 5 Pa, a sputtering power of 80 W, and an Ar flow rate of 40 sccm. The film thickness was approximately 200 nm. To fabricate the MSM detectors, a 50 nm Au film was deposited on the a-IGO film by thermal evaporation. Then, the Au film was lithographed with a mask. As shown in Figure 1a,b, the length of the fabricated detector electrodes was 500 μm, and the finger spacing and width were 5 and 10 μm, respectively.

The thickness was estimated by a surface profile scanner using the steps between film and the substrate. The structure and orientation of the film were tested by D/max-RA X-ray diffraction (XRD and GI-XRD, RIGAKU SmartLab). The film surface roughness and resistance distribution were determined by atomic force microscopy (AFM, Bruker Dimension ICON) and conductive atomic force microscopy (CFM) measurements. The In content and distribution of the films were characterized by energy-dispersive X-ray spectroscopy (EDX, HITACHI SU-70). The transmission rate was tested using a Shimadzu UV-2450PC scanning spectrophotometer, and the detection range and bandgap were also calculated. The important responsivity and response speed parameters are shown from Zolix Solar Cell Scan 100 measurement system (200 W UV-enhanced Xe lamp with a monochromator and Keithley 2450) and transient response test system.

## 3. Results and Discussion

**Figure 2:**
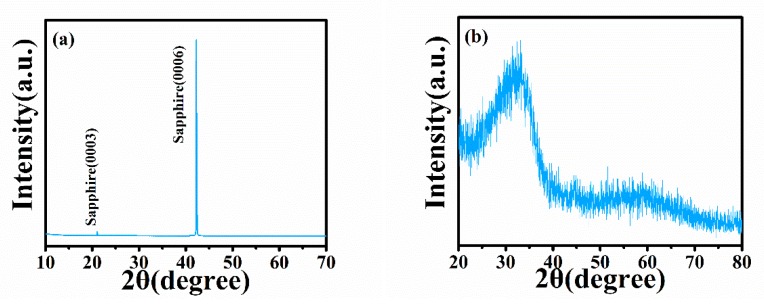
(**a**) Normal XRD spectra and (**b**) XRD grazing incidence (X-ray angle 0.8°) spectra of the a-InGaO thin film.

**Figure 3:**
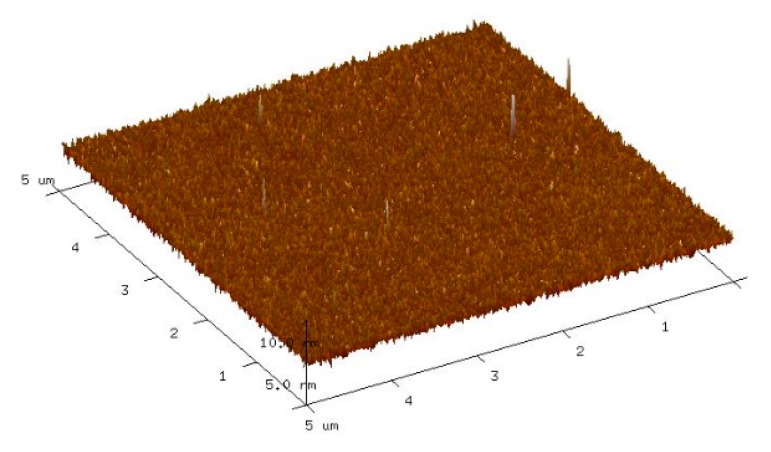
AFM image of the a-IGO thin film surface.

Figure 2a shows the normal XRD spectra of the IGO thin film. From the normal XRD results, there are only two diffraction peaks, namely, (0003) and (0006), that belong to sapphire. To prove that the IGO thin film is amorphous, the characterization of the XRD grazing incidence is necessary. Figure 2b shows the grazing incidence XRD results of the IGO thin film. No obvious peak could be found, which indicates that the film is amorphous. We can obtain the film surface information from AFM. Figure 3 is the AFM image with a 5 μm × 5 μm scanning area. The a-IGO film is very smooth and exhibits a small roughness of ~1 nm, which is consistent with the amorphous characteristic of the film.

**Figure 4:**
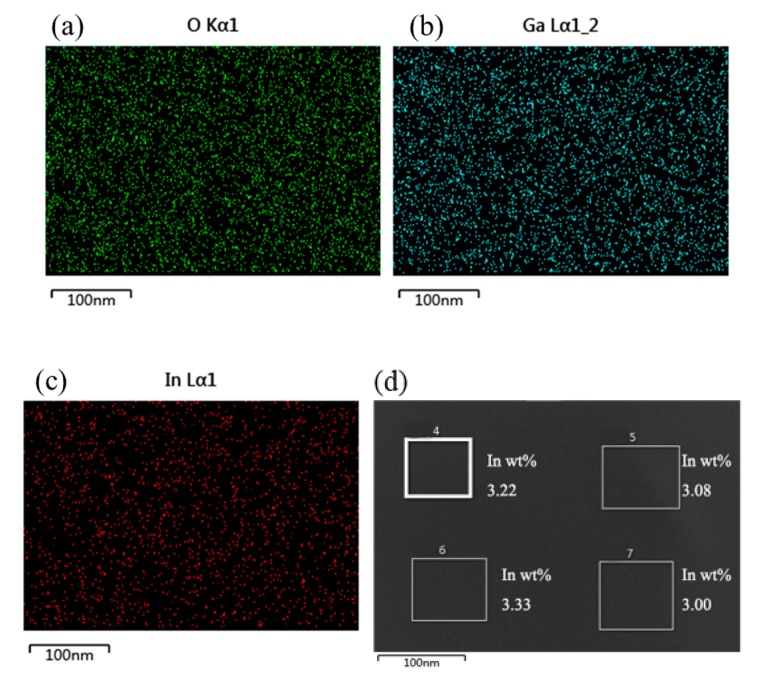
Elemental maps via EDX: (**a**) O; (**b**) Ga; and (**c**) In. (**d**) Small area scanning of the four different areas.

EDX analysis was performed to evaluate the In, Ga, and O contents and distributions in the a-IGO thin film, as shown in Figure 4, with highlight points. In was successfully doped in Ga_2_O_3_; the In element content was 3.48%, the Ga element content was 10.94%, and the O content was ~85.58%. From Figure 4a,b, the distributions of Ga and O elements are even. There are some obvious dark areas where there are no In elements, as shown in Figure 4c. Figure 4d depicts the scanning results of four small areas, and the results indicate that the contents of the different areas are clearly different, which indicates that the In distribution is uneven.

**Figure 5:**
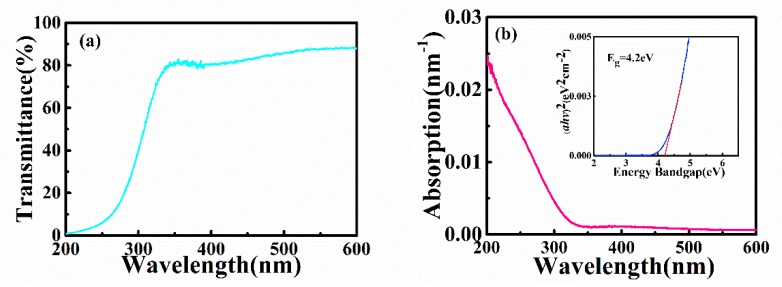
(**a**) The transmission spectrum of the a-IGO thin film. (**b**) The absorption spectrum of the film; the inset shows the plot of (*αhv*)^2^ versus energy bandgap.

Figure 5a is the transmission spectrum of the a-IGO thin film, and the average transmittance in the visible light range is near 85%. For UV light, the transmittance decreases rapidly below 280 nm, and it is close to zero at 200 nm, which indicates that the rate is very low in the range of UVC light. The absorption coefficient can be given by the relation *T* = *Aexp*(−*αd*); *d* is the film thickness, *A* is a constant, *T* is the transmittance [21], and the result is shown in Figure 5b. As the wavelength increases, the absorption rate decreases rapidly. The absorption rate decreases to near zero at approximately 320 nm, which is the absorption cutoff edge of this film. The bandgap *E_g_* can be estimated by the equation (*αhv*)^2^ = *B*(*hv* − *Eg*); *B* is a constant, *hv* is the photo energy [22]. As shown in the inset of Figure 5b, *E_g_* can be extracted from a linear extrapolation of (*αhv*)^2^; the photon energy at the point where (*αhv*)^2^ = 0 is *E_g_*. The obtained *E_g_* is ~4.2eV; it decreases compared to ~4.9eV of Ga_2_O_3_, which is consistent with references [23,24,25]. 

**Figure 6:**
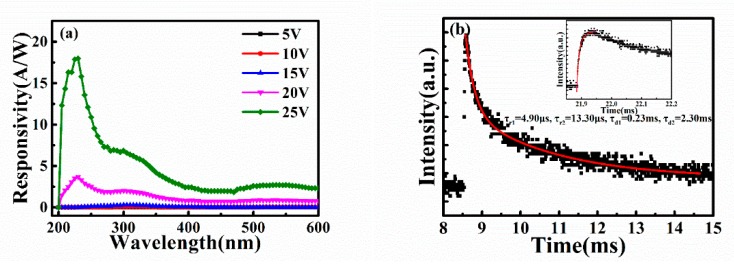
(**a**) The responsivity spectra and (**b**) the time-dependent photoresponse curve of the a-IGO photodetector under 255 nm illumination.

Above the a-IGO thin film, we fabricated Au finger electrodes by lithography to be a photodetector. Figure 6a shows the responsivity under different voltage conditions. The responsivity increases with increasing voltage. The highest responsivity reaches 18.06 A/W under 25 V at 235 nm, which is between the solar-blind wave range. The photodetector also has a larger detection range, which reveals the changeable bandgap because of In doping and proves that the bandgap of Ga_2_O_3_ decreased. The test range is broadened to 210–330 nm, which is wider than the individual Ga_2_O_3_ photodetector.

As a photodetector, the response speed is a key parameter. Figure 6b exhibits the response time curves of the detector. When the detector was illuminated by the 255 nm light, the photocurrent increased rapidly. After the light was turned off, the detector needs time, which is decay time (longer than the rise time), to recover to the dark current. To obtain the specific numbers of the rise time and decay time, the response curves were fitted, and these constants were analyzed by the double exponential equation:

$$y=y_{0}+y_{1}\exp\left(-t/\tau_{1}\right)+y_{2}\exp\left(-t/\tau_{2}\right).$$

In this equation, *y*_0_ is the steady-state dark current, *y*_1_ and *y*_2_ are both constants, *t* refers to the time, and $\tau_{1}$ and $\tau_{2}$ stand for the relaxation time, including the fast and slow two stages of the rise time ($\tau_{r}$) and decay time ($\tau_{d}$). From the well-fitted curves, the rise time (*τ_r_*_1_/*τ_r_*_2_ = 4.9 μs/13.3 μs) and decay time (*τ_d_*_1_/*τ_d_*_2_ = 0.23 ms/2.3 ms) results are faster than those of the other Ga_2_O_3_-based solar-blind photodetectors listed in Table 1.

**Figure 7:**
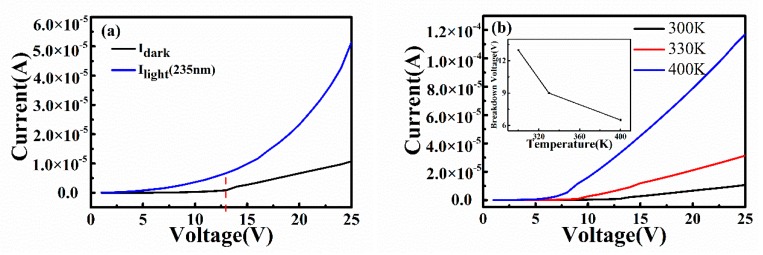
**(a)** The dark and light I–V curves of a-IGO photodetector and (**b**) variable temperature I–V curves without light; the inset shows the relationship between breakdown voltage and temperature.

As previously mentioned, the speed of the device response and recovery are greatly reduced because of the persistent photoconductive effect of the β-Ga_2_O_3_ thin film detector [19]. However, our photodetector both has fast response speed and high responsivity. Therefore, the a-IGO photodetector may have a different response mechanism than the crystallization photodetector. The current–voltage (I–V) characteristic is shown in Figure 7a with dark current and photocurrents, which were measured at 235 nm. As shown in the Figure 7a, the dark current below ~13 V is small and increases significantly above ~13 V. There is an obvious kink point at ~13 V, which implies that the device has a breakdown voltage of ~13 V at room temperature. There may be two recoverable breakdown internal gains, an avalanche gain mechanism or a Zener tunneling effect. To determine what the internal gain type is, we tested the dark current at changeable temperatures (I–V curves at different temperatures are shown in Figure 7b and the inset figure). The tunnel breakdown voltage decreases from 13 V under 300 K, which is close to room temperature to 6.5 V under 400 K. Through a calculation, the temperature coefficient is negative, −0.065 V/K, which indicates that the gain in our a-IGO photodetector could be a quasi-Zener tunneling effect.

**Figure 8:**
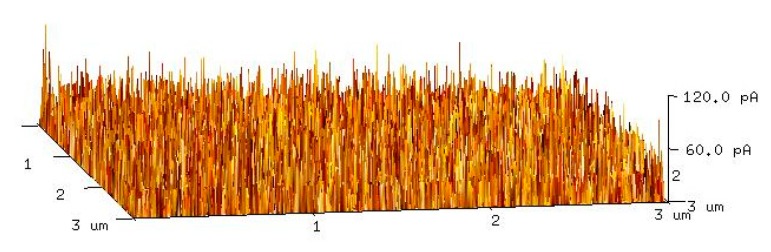
The CFM result of the dark current distribution under a 5 V bias voltage.

The breakdown phenomenon always occurs in some types devices, such as the avalanche photodetector (APD), metal–insulator–semiconductor (MIS) tunnel junction, and the p–n junction [31,32,33]. The quasi-Zener tunneling effect that exists in our detector indicates that there may be different resistance areas between which barriers exist. CFM tests were carried out to determine the surface dark current density. As shown in Figure 8, the resistance distribution in the a-IGO thin film is not uniform, and the resistance values of different regions are different. This phenomenon may be due to the uneven distribution of In, which can be seen in Figure 4c. There are some obvious dark areas in the In distribution mapping. Figure 4d, which is the small area scanning of EDX, shows that the In contents are different at different areas in the film. The low resistance regions have high In content, and the high resistance regions have low In content [34]. The thin high resistance regions are shown in Figure 8.

When a small bias voltage is applied to both ends of the electrode, most of the carriers cannot jump over the barrier formed by the high resistance region. Carriers are blocked by a distributed small area of high resistance, and I_dark_ of the a-IGO detector is relatively smaller. As the bias voltage increases to the breakdown voltage, a large number of carriers in the low resistance region can break through the high resistance region by tunneling which usually occurs in MIS-structure tunnel junction detectors [33], and an internal gain mechanism appears. The formula for the carrier tunneling probability is:

$$T_{t}=\exp\left(-\frac{2d\sqrt{2qm^{*}\phi_{T}}}{\hbar }\right).$$

Here, q, $m^{*}$ and ℏ are constants, $\phi_{T}$ represents the barrier of the high resistance region, and *d* represents the distance of tunneling. The distance *d* can be seen as the effective thickness, and the probability of tunneling *T_t_* increases exponentially with $\phi_{T}$ and *d* decreasing. When the device is exposed to deep ultraviolet light, the photogenerated carriers appeared in both high resistance areas and low resistance areas. The photogenerated carriers drop the effective thickness and barrier of the high resistance regions, and also increase the carrier concentration of the low resistance regions. Therefore, the carrier tunneling probability increases, and the photocurrent increases exponentially and is much higher than the dark current. The switching between the above block process and tunneling process is very quick, which has been reported in MIS-structure tunnel junction detectors [35]. So our a-IGO photodetector with quasi-Zener tunneling effect exhibits faster response and recovery speed compared to other crystallization devices with persistent photoconductive effect.

## 4. Conclusions

In summary, the In-doped Ga_2_O_3_ amorphous thin film was successfully deposited on sapphire by RF sputtering at room temperature. A fast response MSM solar-blind photodetector with a higher responsibility of 18.06 A/W under a 25 V bias voltage was fabricated based on the film. The rise time and decay time reached 4.9 and 230 μs, respectively. The uneven In distribution results in the quasi-Zener tunneling effect improved the photodetector’s key parameters. Simultaneously, In doping changed the bandgap of the films, and thus, the detection range of the photodetector was broadened from 210 to 330 nm. The high-performance a-IGO solar-blind photodetector preparation at room temperature is very important for flexible devices; additionally, the process is inexpensive and can be developed in for applications in different fields. 

## Figures and Tables

**Table 1:** Comparison of key parameters of the different solar-blind photodetectors.

Material	Structure	R [AW^−1^]	*τ_r_*_1_/*τ_r_*_2_	*τ_d_*_1_/*τ_d_*_2_	Ref.
a-Ga_2_O_3_	thin film MSM	70.26 @ 20 V	0.41/2.04 s	0.02/0.35 s	[18]
a-Ga_2_O_3_	thin film MSM	0.19 @ 10 V	19.1 μs	80.7 μs	[19]
β-Ga_2_O_3_	thin film MSM	259 @ 20 V	2.1 s	0.4 s	[26]
β-Ga_2_O_3_	thin film MSM	96.13 @ 5 V	32.2 ms @ 0 V	78 ms @ 0 V	[27]
β-Ga_2_O_3_	thin film MSM	3.3 @ 16 V	3.33 s @ 20 V	0.4 s @ 20 V	[28]
Zn: β-Ga_2_O_3_	thin film MSM	210 @ 20 V	3.2 s	1.4 s	[29]
Si: β-Ga_2_O_3_	thin film MSM	1.45 @ 5 V	0.58/32.93 s	1.2/32.86 s	[30]
InGaO	nanobelt	547 @ 40 V	1 s	0.6 s	[23]
InGaO	thin film MSM	0.31 @ 10 V	21 s	27 s	[24]
a-InGaO	thin film MSM	6.9 × 10^−5^ @ 5 V	2.4/0.4 s	18.2/0.4 s	[25]
a-InGaO	thin film MSM	18.06 @ 25 V	4.9/13.3 μs	0.23/2.3 ms	Our work
